# Additional effect of pain neuroscience education to craniocervical manual therapy and exercises for pain intensity and disability in temporomandibular disorders: a study protocol for a randomized controlled trial

**DOI:** 10.1186/s13063-021-05532-x

**Published:** 2021-09-06

**Authors:** Aroldo dos Santos Aguiar, Cesar Bataglion, Lilian Ramiro Felício, Beatriz Azevedo, Thaís Cristina Chaves

**Affiliations:** 1 Graduate Program on Rehabilitation and Functional Performance, Ribeirão Preto Medical School, University of São Paulo, Ribeirão Preto, São Paulo, Brazil; 2 Department of Restorative Dentistry at Ribeirão Preto Dental School (FORP), University of São Paulo-USP, Ribeirão Preto, São Paulo, Brazil; 3 Graduate Program of Physical Therapy, Laboratory of Evaluation in Biomechanics and Neurosciences (LABiN), Federal University of Uberlândia, Uberlândia, Minas Gerais Brazil; 4 Department of Health Sciences and Graduate Program on Rehabilitation and Functional Performance, Laboratory of Research on Movement and Pain (LabMovePain), Ribeirão Preto Medical School, University of São Paulo, Ribeirão Preto, São Paulo, Brazil

**Keywords:** Temporomandibular joint disorders, Patient education, Exercise therapy, Musculoskeletal manipulations, Chronic pain, Disability evaluation

## Abstract

**Abstract:**

The objective of this study will be to investigate the additional effect of pain neuroscience education program compared to a craniocervical manual therapy and exercises program for pain intensity and disability in patients with temporomandibular disorders (TMD). This study will be a randomized controlled trial comprising a sample of 148 participants. Subjects between 18 and 55 years, both genders, will undergo a screening process to confirm painful TMD by the Research Diagnostic Criteria (RDC/TMD), and then the volunteers will be randomized into two groups (G1: pain neuroscience education + craniocervical manual therapy and exercises vs. G2: craniocervical manual therapy and exercises). The volunteers will be recruited at the dentistry clinic. The intervention will be administered twice a week for 6 weeks by a single therapist lasting 1 h per session. The primary outcome will be pain intensity and disability and the secondary outcomes will be pain self-efficacy, kinesiophobia, and global perceived effect of improvement. The participants will be assessed immediately after the last session and at one- and three-month follow-ups. All statistical analyses will be conducted following intention-to-treat principles, and the treatment effects will be calculated using linear mixed models. The results of this study may contribute to understand the additional effect of pain neuroscience education intervention on TMD patients submitted to manual therapy and exercise.

**Trial registration:**

ClinicalTrials.gov NCT03926767. Registered on April 29, 2019.

## Background

Temporomandibular disorders (TMD) is a collective term that encompasses several clinical diagnoses involving the masticatory muscles, temporomandibular joints (TMJs), and associated structures [1]. The literature recognizes that TMD is not caused by a single factor, it is a complex disorder associated with comorbidities, physical signs and symptoms, changes in behavior, emotional status, social interactions, and changes in the function and structure of the central nervous system [2, 3].

TMD is considered the most frequent cause of chronic orofacial pain of non-dental origin, with a point prevalence of 10-15% for adults [4, 5]. In addition, there is a coexistence between TMD and neck pain [6–8], and interventions focused on the craniocervical region as a whole may contribute to decrease pain [9].

Since painful TMD has a multifactorial pathophysiology [2], there is no single approach for treating patients with TMD and the effective management of such disorder has not been established yet [10]. Several studies have demonstrated the effectiveness of interventions like manual therapy associated or not to therapeutic exercise for pain intensity and disability for TMD [9–11]. Greater effect on pain intensity for interventions focused on both orofacial and neck region against placebo/minimal intervention have been reported [9]. Most of the studies included in such systematic reviews showed small sample sizes or adopted a comparison group not submitted to an active treatment.

Current literature shows compelling evidence that the mechanistic biomedical model is not suitable to manage patients with chronic painful TMD [12]. There is a movement towards treatment modalities that encompass the biopsychosocial model that acknowledges and aims to address the biological (physical) and psychosocial factors to treat chronic pain [13, 14].

Therapeutic patient education intervention comprises the provision of information to improve the patient's understanding of their problem, speeding up the return to activities and minimizing the dependency of health professionals [15]. Pain neuroscience education (PNE) consists of a set of cognitive interventions whose main objective is to change the patient's conceptualization about pain [16]. A systematic review reported that the combination of PNE with other interventions resulted in more favorable responses for pain intensity, disability and pain catastrophizing in patients with chronic musculoskeletal disorders [17]. Moreover, pain education is the first-line recommendation in clinical practice guidelines for the management of musculoskeletal pain [18, 19].

There is no previous study published in the literature investigating the additional effect of PNE to a protocol of craniocervical manual therapy and exercises for patients with TMD. Then, the objective of our study will be to evaluate the additional effect of PNE to craniocervical manual therapy and exercises on pain intensity and orofacial disability immediately and after 1-month and 3-month follow-ups in patients with TMD. Also, as secondary outcomes, we will investigate the effect of the protocol to pain self-efficacy, kinesiophobia, and global perceived effect of improvement. The hypothesis of this study is that patients submitted to both PNE and manual therapy and exercises will show better outcomes for pain intensity and orofacial-related disability than patients submitted to manual therapy and exercises alone.

## Methods

### Trial design

This study will be a randomized clinical trial with two parallel arms, following the recommendations of the Consolidated Standards of Reporting Trials - CONSORT [20].

### Approval and registration

The study was submitted to and approved by the ethics committee for research involving human subjects of the Clinics Hospital of the Ribeirão Preto, Medical School of the University of São Paulo (HCFMRP Process N° 3449/2018). The study was registered prospectively on Clinical Trials (NCT03926767).

### Eligibility criteria and participants

A sample size of 148 female and male patients with painful TMD will participate in this study. They will be consecutively recruited from the Orofacial Pain Outpatient Clinic from the School of Dentistry of Ribeirão Preto, University of São Paulo. The study is under recruitment and the estimated study completion date is on February 2022. The inclusion criteria for participants were as follows: (i) A diagnosis of painful TMD according to Research Diagnostic Criteria for TMD (RDC/TMD) [21], (ii) history of orofacial pain at least three months prior to the study [22] and (iii) age ranging between 18 and 55 years, considering the greater prevalence associated with this age period [23].

Illiterate patients, severe depression (medical diagnoses), clinical history of tumors in the craniofacial region, patients in the post dental surgery period or submitted to previous physical therapy in the past year or to any health/pain education strategy, pregnant women, infections, whiplash-associated disorders and with chronic degenerative inflammatory or neurologic disorders were excluded from this study. Patients will be instructed to not use pain relief medications during the intervention period of this trial and if any medication be used, participants will be encouraged to report. All participants will be informed about the procedures of this study, will have to report agreement to participate, and will sign the consent form. Participants will be instructed that they will be free to remove consent at any time of the study.

### Analysis of the population

In the event of two consecutive absences from treatment sessions, patients will be contacted by telephone. The analysis will follow the intention-to-treat principles. An intention-to-treat analysis will be performed using the patient’s most recent assessment in case of withdrawals or absence of data. Possible adverse effects (injuries) occurring during the intervention period, the individuals will be referred for appropriate treatment.

### Procedures, randomization, and allocation

**Fig 1:**
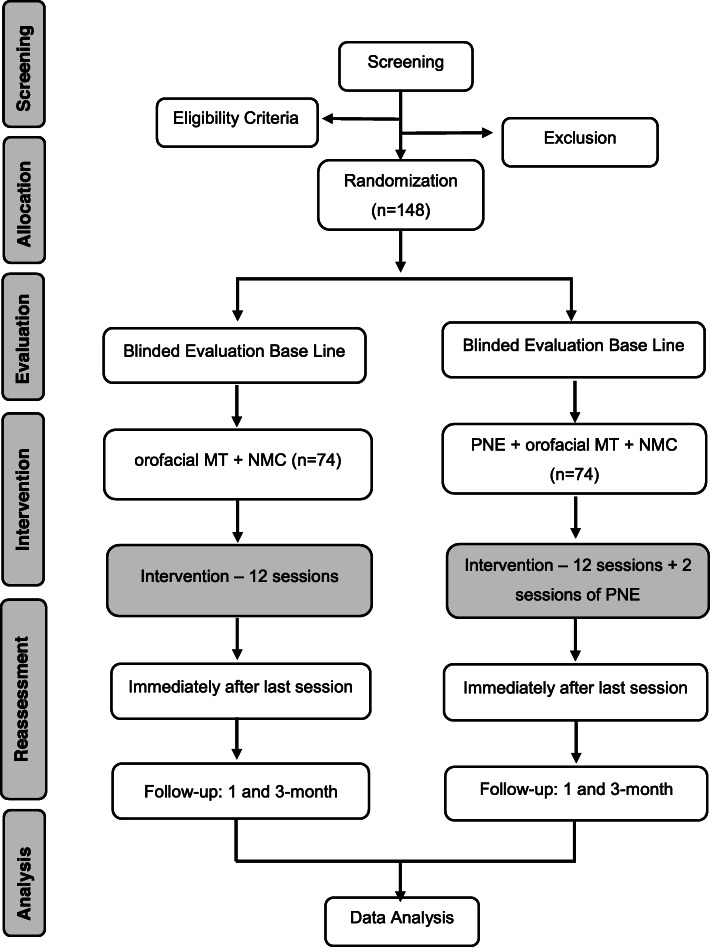
Flow chart demonstrating the randomization process to be adopted in the present study. PNE, pain neuroscience education; MT, manual therapy; NMC, neck motor control exercises

Once the patient has accepted the invitation to participate and signed the formal consent to participate, one researcher will run the assessment to determine eligibility. After this initial assessment, participants will be randomly assigned following simple computerized randomisation procedures to one of the two treatment groups through the use of cards previously placed in opaque sealed envelopes: G1 – Pain Neuroscience Education + craniocervical manual therapy and exercises or G2 - craniocervical manual therapy and exercises. The allocation sequence will be generated by a researcher not involved in the assessment and interventions (TCC), and another research assistant will assign participants to interventions (Fig. 1). Figure 1 shows the trial procedure, and Table 1 illustrates the trial schedule.

**Table 1:**
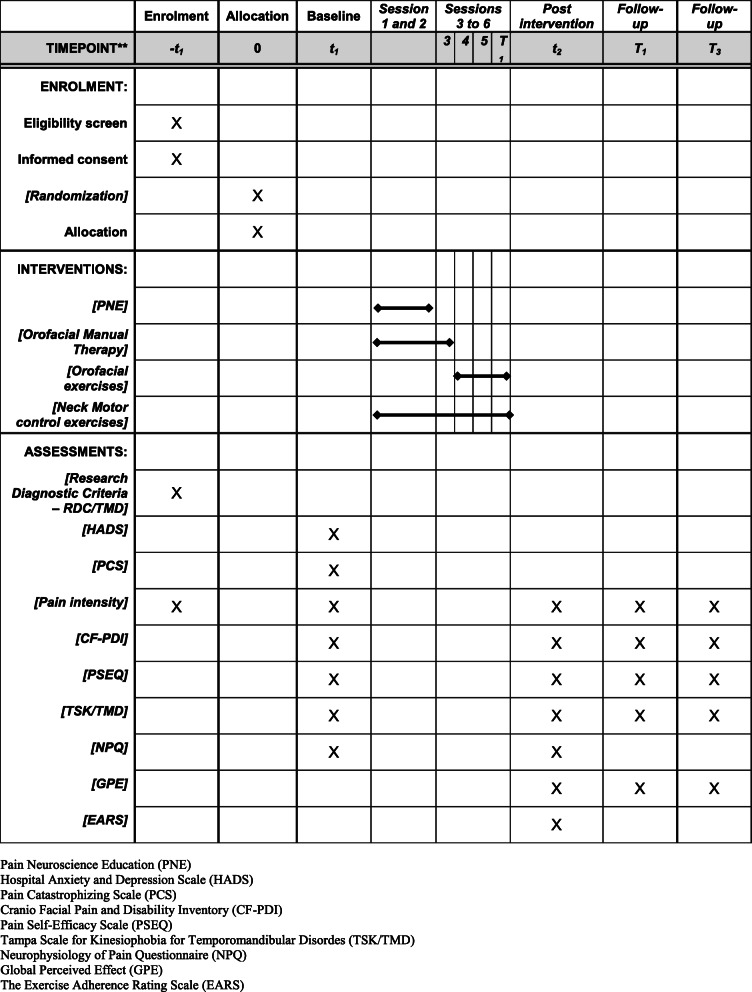
Recommended protocol items: schedule for enrollment, interventions, and assessments

*PNE* = pain neuroscience education, *HADS* = Hospital Anxiety and Depression Scale, *PCS* = Pain Catastrophizing Scale, *CF-PDI* = Cranio Facial Pain and Disability Inventory, *PSEQ*= Pain Self-Efficacy Scale, *TSK/TMD* = Tampa Scale for Kinesiophobia for Temporomandibular Disordes, *NPQ =* Neurophysiology of Pain Questionnaire, *GPE* = Global Perceived Effect, *EARS* = The Exercise Adherence Rating Scale

One blinded researcher regarding treatment group allocation will run the assessments pre-treatment, immediately after and at 1-month and 3-month follow-ups. The evaluations of the study will be carried out according to the recommendations of the Initiative on Methods, Measurement and Pain Assessment in Clinical Trials (IMMPACT) for chronic pain [24].

During the assessment for eligibility, the participants will be assessed according to RDC/TMD to confirm the presence of TMD [21]. Afterwards, the participants will answer the Pain Numeric Rating Scale (NPRS) [25], The Craniofacial Pain and Disability Inventory (CF-PDI) [26], Global Perceived Effect (GPE) of improvement [25], Tampa Scale for Kinesiophobia for Temporomandibular Disorders (TSK/TMD )[27], Pain Catastrophizing Scale (PCS) [28], Pain Self-Efficacy Questionnaire (PSEQ) [29], Revised Neurophysiology of Pain Questionnaire (NPQ) [30], and Hospital Anxiety and Depression Scale (HADS) [31]. This protocol followed the Standard Protocol Items: Recommendations for Interventional Trials (SPIRIT) recommendations [32].

### Baseline assessment

#### Hospital anxiety and depression scale (HADS)

HADS [33] will be employed to identify anxiety disorders and depression. It was translated and validated into Brazilian Portuguese. The HADS is divided into the anxiety subscale (HADS-A) and the depression subscale (HADS-D), both containing seven interspersed items. It is composed of seven items for depression and seven items for anxiety, each item including four response options ranging from 0 to 3. A cutoff of ≥8 was described with good sensitivity and specificity values (0.70–0.90) for anxiety and depression symptoms [31].

#### Pain Catastrophizing Scale (PCS)

The PCS Brazilian Portuguese [28] is a self-administered questionnaire that consists of 13 items for the assessment of catastrophizing thoughts. It is divided into three domains: helplessness, magnification, and rumination. Each item is scored on a 5-point ordinal scale. The B-PCS total score ranges from 0 to 52 points, higher values denote greater pain catastrophizing. Acceptable values for validity, internal consistency, and test-retest reliability are described for the Brazilian PCS [28].

### Primary outcomes

#### Pain intensity

The NPRS [25] will be used to assess pain intensity in this trial and consists in a sequence of numbers from 0 to 10, in which 0 represents “no pain” and 10 represents “worst pain imaginable” [25].

#### Orofacial and pain disability

The Craniofacial Pain and Disability Inventory (CF-PDI) [34] is a self-administered questionnaire that measures the outcomes of pain and disability related to craniofacial pain and demonstrated an acceptable structural validity, internal consistency, reproducibility, and construct validity [34]. Also, the Brazilian Portuguese version showed acceptable measurement properties [26]. It consists of 21 items, with a score ranging from 0 to 63 points. Each question is scored on a 4-point ordinal scale, ranging from 0 to 3. A higher score reflects higher disability levels.

### Secondary outcome measures

#### Pain Self-Efficacy Questionnaire -PSEQ

Study participants will be evaluated on self-efficacy related to chronic pain, which can be defined as an individual’s confidence he/she can successfully produce desirable results related to living with chronic pain. The PSEQ has 10 items which are rated on a 7-point ordinal scale (ranging from 0: “*not at all confident*” to 6: “*completely confident*”). It was adapted and validated to Brazilian Portuguese [29]. Previous research showed an effect on self-efficacy using a PNE intervention based on metaphors compared to an intervention using cognitive-behavioral concepts [35].

#### Tampa Scale for Kinesiophobia for Temporomandibular Disorders - TSK/TMD

The TSK/TMD is a self-report questionnaire that assesses the fear of movement [36]. In this study, the TSK-TMD with 12 items validated in Brazilian Portuguese was used [27] and showed acceptable psychometric measurements. Each item is scored on a 4-point ordinal scale, ranging from “strongly disagree” (score = 1) to “strongly agree” (score = 4). Ratings are summed to yield a total score. Higher scores reflect a greater fear of movement (12–48 points).

### Participant ratings of Global Perceived Effect - GPE

The GPE of improvement used for this trial is an 11-point scale that ranges from − 5 (“vastly worse”) through 0 (“no change”) to + 5 (“completely recovered”) and participants are asked: “*Compared to when this episode first started, how would you describe your orofacial pain these days*?”. A higher score indicates higher perception of recovery from the condition [25].

#### The revised Neurophysiology of Pain Questionnaire - NPQ

The NPQ is a self-administered instrument to assess the knowledge regarding pain neurophysiology. The questionnaire will be administered to quantify the level of knowledge of the participants after the PNE program. Each item has the following response options: true (1 point), false (0 point), and undecided (0 point). The maximum score is 12 points in the revised version. The NPQ showed acceptable internal consistency and good test-retest reliability [37]. There is a cross-culturally version of the NPQ to Brazilian Portuguese [30].

#### The Exercise Adherence Rating Scale - EARS

EARS is a patient-reported outcome measure composed of six items that directly assess adherence behavior. In the present study, it will be used to assess the perception of adherence to prescribed home exercises. It was translated and validated into Brazilian Portuguese [38]. The six items should be summed and items with positive phrases are reversely scored, meaning items 1, 4, and 6. The six items are scored using an ordinal Likert scale of possible answers (0 = strongly agree to 4 = totally disagree), with higher scores indicating greater adherence (0 to 24). Participants will answer the EARS only at the end of the treatment, to assess retrospectively the adherence behavior.

### Interventions—general procedure

On both groups, participants will be submitted to craniofacial manual therapy and an exercise program comprised of Orofacial Exercises and Neck Motor Control Exercises. The participants will be submitted to a program of six weeks of craniofacial manual therapy, orofacial exercises, and neck motor control exercises. The program will be carried out twice a week, one session in the outpatient clinic and another day in the week, the participants will be invited to perform home-based exercises.

Each session will last one hour, always conducted by the same physiotherapist. The first three sessions, participants will be submitted to orofacial manual therapy techniques (myofascial release) and neck motor control exercises (Table 2). And in the remaining sessions, participants will perform half the session orofacial exercises and the other half, neck motor control exercises (Table 2). In the first three weeks, only neck motor control exercises (cervical bracing in different positions) will be home-based prescribed. In the last 3 weeks, participants will be instructed to perform orofacial exercises and neck motor control exercises (Table 2).

**Table 2:** The schedule of the interventions administered in the study. The cervical motor control exercises will be administered according to the progression of the patient in each level

		Orofacial manual therapy and exercises	Neck motor control exercises	Pain education
**Week 1**	Outpatient clinic	Myofascial release (Fig. 2)	Cervical bracing (Fig. 4) Neck dynamic isometric exercises (Fig. 5) Neck functional exercises (Fig. 6)	Pain neuroscience education^a^
	Home exercises		Cervical bracing (Fig. 4)	
**Week 2**	Outpatient clinic	Myofascial release (Fig. 2)	Cervical bracing (Fig. 4) Neck dynamic isometric exercises (Fig. 5) Neck functional exercises (Fig. 6)	Pain neuroscience education^a^
	Home exercises		Cervical bracing (Fig. 4)	
**Week 3**	Outpatient clinic	Myofascial release (Fig. 2)	Cervical bracing (Fig. 4) Neck dynamic isometric exercises (Fig. 5) Neck functional exercises (Fig. 6)	
	Home exercises		Cervical bracing (Fig. 4)	
**Week 4**	Outpatient clinic	Mandibular exercises (Fig. 3)	Cervical bracing (Fig. 4) Neck dynamic isometric exercises (Fig. 5) Neck functional exercises (Fig. 6)	
	Home exercises	Mandibular exercises (Fig. 3)	Cervical bracing (Fig. 4)	
**Week 5**	Outpatient clinic	Mandibular exercises (Fig. 3)	Cervical bracing (Fig. 4) Neck dynamic isometric exercises (Fig. 5) Neck functional exercises (Fig. 6)	
	Home exercises	Mandibular exercises (Fig. 3)	Cervical bracing (Fig. 4)	
**Week 6**	Outpatient clinic	Mandibular exercises (Fig. 3)	Cervical bracing (Fig. 4) Neck dynamic isometric exercises (Fig. 5) Neck functional exercises (Fig. 6)	
	Home exercises	Mandibular exercises (Fig. 3)	Cervical bracing (Fig. 4)	

^a^Pain education was administered just for one group

Pain neuroscience education will be administered only to one group in the first and second sessions (half of the session). To balance the clinical attention offered to patients in both groups, the group not submitted to PNE will be invited to clarify possible doubts regarding the home exercises.

#### The Orofacial Exercises and Orofacial Manual Therapy

**Fig. 2:**
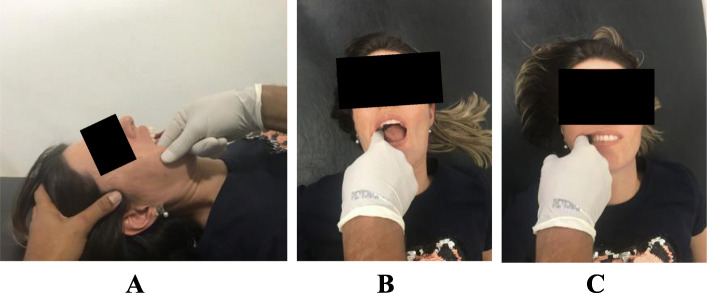
The manual therapy techniques. **A** Intraoral temporalis myofascial release: The therapist will be positioned by the side in which the technique will be administered, one hand will be on the coronoid process of the mandible with pressure according to the patient's tolerance, while the other hand will be along the temporal muscle. The patient will perform opening movements of the mouth gradually until the maximum opening. **B** Intraoral medial and lateral pterygoid (origin) technique: The therapist will be positioned contralateral by the side in which the technique will be administered, and with the index finger, the therapist will press the origins of the pterygoid muscles, the pressure will be carefully maintained for 5 s. **C** Intraoral sphenopalatine ganglion technique: The therapist's index finger will be slowly inserted along the buccal surface of the slightly occluded teeth, the patient will tighten the teeth and, after relaxing, the therapist will apply pressure behind the lingual surface of the masseter and medial pterygoid, this process is repeated until the fingertip approaches the anterior surface of the infratemporal fossa/sphenopalatine fossa, in a comfortable way for the patient

**Fig. 3:**
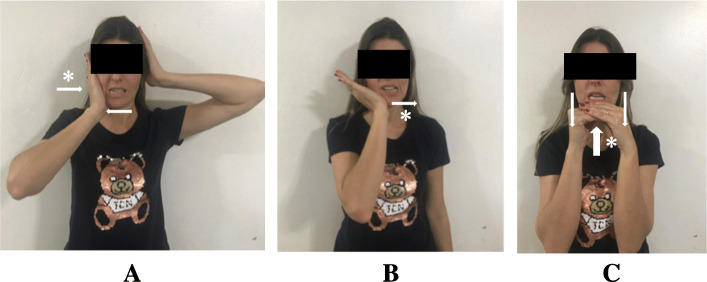
Mandibular exercises. (**A**) Mandibular body - condylar cross-pressure chewing technique. Post-isometric relaxation stretches – jaw laterotrusion (**B**) and jaw-opening (**C**). Arrows indicate the direction of the movements. * Direction of the movement of the hand

The protocol of Orofacial Exercises and Manual Therapy reported by Kalamir et al. [39] will be adopted in the present study. The manual therapy techniques: intraoral temporalis muscle release, intraoral medial and lateral pterygoid (origin) muscles technique, and intraoral sphenopalatine ganglion technique (Fig. 2). And two mandibular exercises: Mandibular body—condylar cross-pressure chewing technique and post-isometric relaxation stretches in jaw laterotrusion and jaw opening (Fig. 3). Each exercise will be executed 10 times per session for 10 s. Participants gave written consent for use of his/her images on pictures 2 to 6.

#### Neck motor control exercises

The neck motor control protocol reported by Celenay et al. [40] will be adopted in our study. The exercise program has three levels: (1) *cervical bracing*, (2) *neck dynamic isometric exercises*, and (3) *neck functional exercises*.

**Fig. 4:**
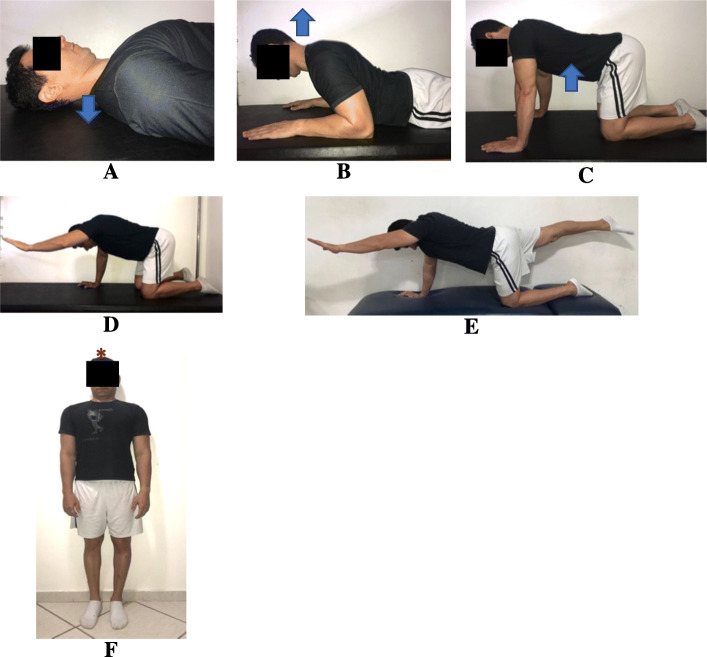
Neck motor control exercises program. **A**–**C** Cervical bracing on neurodevelopment stage positions. **D**, **E** The cervical bracing with extremity range-of-motion movements. **F** Cervical bracing in the standing position—movement against the wall. Arrows indicate the direction of the movements. * Direction of the movement against the wall

The *cervical bracing exercises* include four hierarchical levels in neurodevelopment stages (supine, prone, quadrupedal, bipedal) for the cervical spine (Fig. 4). The intermediate level exercises will be comprised of upper and lower extremity range of motion exercises while maintaining a stable spine in four-, three-, or two-point kneeling (Fig. 4C–E). The high-level exercise will be the cervical bracing in the upright position (Fig. 4F).

**Fig. 5:**
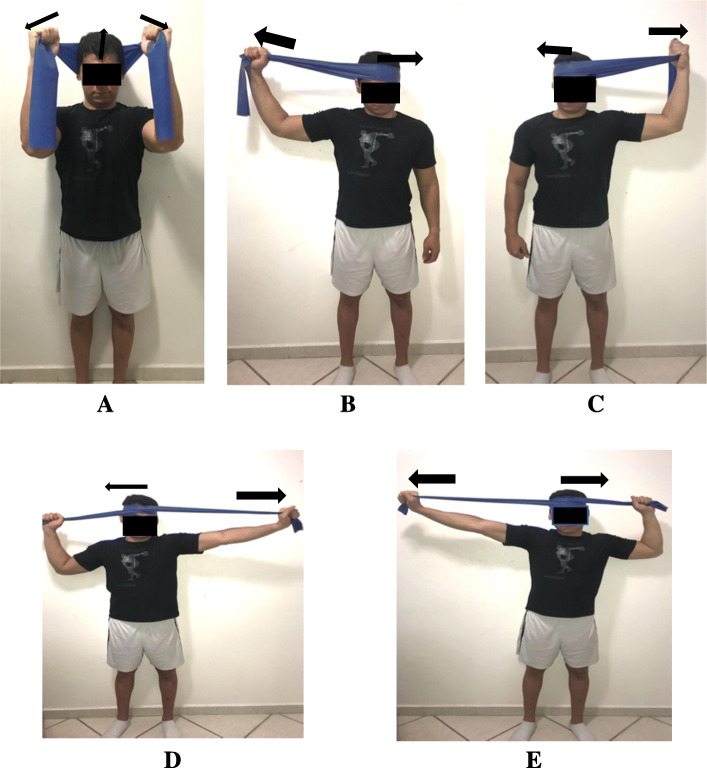
Neck motor control exercises program—neck dynamic isometric exercises were performed directly forward (**A**), obliquely, toward right (**C**) and left (**B**), and directly backward by maintaining a stable spine with elastic resistive bands (**D**, **E**). Arrows indicate the direction of the movements

The *neck dynamic isometric exercises* (five hierarchical levels) will be carried out directly forward, obliquely, toward right and left, and directly backward by maintaining a stable spine with elastic resistive bands (Fig. 5).

**Fig. 6:**
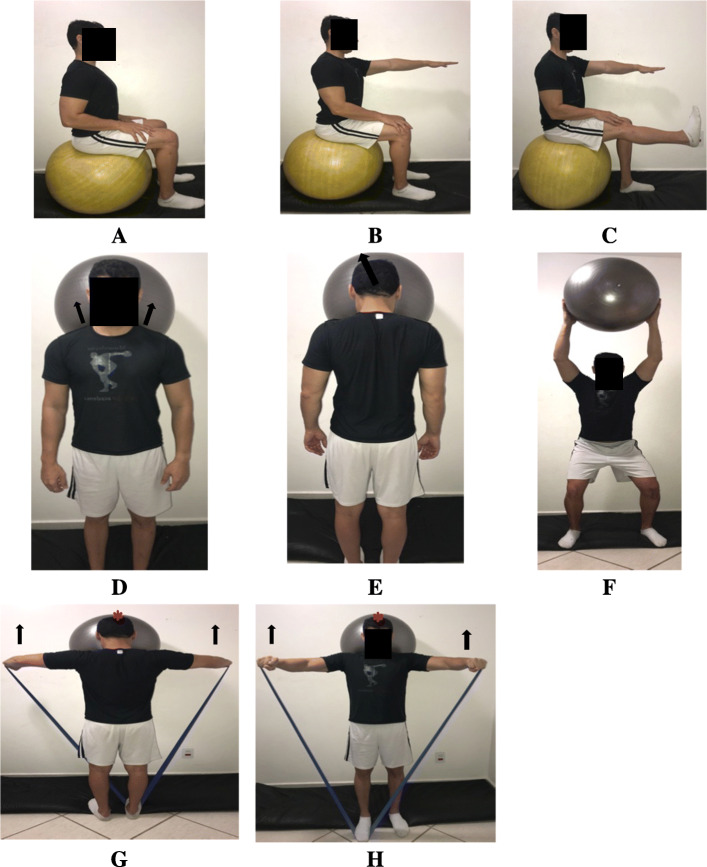
Neck motor control exercises program—functional training on unstable surface was performed in combination with cervical bracing over the ball (**A**–**C**), with ball in standing position (**D**–**F**) and with ball and elastic resistance (**G**, **H**). Arrows indicate the direction of the movements. * Direction of the movement against the wall

Finally, *neck functional exercises* will include functional training with elastic resistance and exercise balls on unstable surfaces (eight hierarchical levels) (Fig. 6A–H). The criteria to progress in each level (cervical bracing, neck dynamic isometric exercises, and neck functional exercises) will be holding the contraction, for 10 s, 10 times. The progression of the exercises adopted will follow the sequence described on Figs. 4, 5, and 6.

Participants will be oriented to contract deep neck flexor muscles only during the exercises, not throughout the day as described in the protocol of the original paper [40].

#### Pain neuroscience education (PNE)

All participants in the G1 will initially receive two additional sessions in which a workshop on PNE will be administered and discussed. A power-point presentation with metaphors and animated videos will be employed. The PNE program will be held in 2 sessions of 40 min each. The intervention program will be divided into 17 thematic topics according to explain pain [41]:

1. Contextualization on the importance of the program: how pain causes suffering and its alarming increase in the world
2. Initial concepts on neurosciences and pain
3. How context can influence pain perception—incredible stories on pain
4. Human beings as a multisensory complex—sensory information is reaching the brain all the time
5. Pain output may be deflagrated by memory, not only nociception
6. Nociception and the concept of pain as a response of protection/the nociceptors
7. The incorrect concepts on pain (pain system, pain receptors, pain area in the brain)
8. Concepts on pain neurophysiology—synapses, spinal horn, primary and secondary order neurons
9. Types of sensitization/descending nociception modulation system
10. The danger message and the brain processing
11. The sensitized brain and its relationship to chronic pain
12. The contribution of other systems to pain experience and vice-versa: endocrine, parasympathetic/sympathetic, immune, and motor
13. How bone, muscles, and nerves send sensory information all the time
14. Fear-avoidance model revisited
15. Encouragement to change
16. How to develop positive attitudes
17. Concepts of graded exposure and graded activity - (at least two questions). We will reconceptualize the beliefs of the questions with higher scores on TSK/TMD. A schedule will be developed together with the patient to favor patient exposition to activities they used to avoid.

### Sample size calculation

The study was designed to detect a between-group difference of 2 points in pain intensity (30%) measured by the NPRS, with an estimated standard deviation of 3 points. A previous study [42] recommended a minimum sample size of n=61 per group.

For disability, a between-group difference of 5.08 points for disability measured by the CF-PDI, considering the Smallest Detectable Change of CF-PDI Brazilian version [26], with an estimated standard deviation of 10 points resulted in a sample size of 64 participants per group. The other specifications were power of 80%, an alpha of 5%, *f* test of 0.33 (pain intensity) and 0.25 (disability). Therefore, a total of 128 participants was obtained, however considering a follow-up loss up to 15%, we will recruit 148 participants. G*Power was used to run the sample size calculation (GPower 3.0.10, da University of Kiel, Germany).

### Data analysis

The mean effects of the interventions and the group differences for all outcomes (primary and secondary) will be calculated using linear mixed models, incorporating terms for the treatment groups, time (post-intervention and follow-up), and interaction terms (treatment subgroups and time) as well as, psychosocial variables, knowledge about pain neurophysiology (score on the NPQ), sex, ethnicity, and age as covariates. Secondary analysis will be conducted using regression models to determine whether baseline scores of psychosocial variables (HADS, PCS, TSK/TMD) will moderate the effect of treatments. All data will be double entered prior to the analysis. The analysis will follow the intention-to-treat principles. Additionally, we will adopt analysis per protocol excluding patients that did not complete the 6 weeks of treatment. The statistician will receive coded data and will be blinded to the participants’ allocation groups. The data collected will be stored coded to protect patient confidentiality. In order do to handle missing data, we will replace the value by the last observation carried forward. For all analysis, we will use the IBM SPSS software package, version 22 (IBM Corp, Armonk, NY) and the significance level will be established at 0.05.

## Discussion

This study will be the first randomized controlled trial in which the additional effect of PNE to Orofacial Manual Therapy/Orofacial exercises/Neck Motor Control exercises will be investigated in patients with chronic TMD pain for the outcomes of pain intensity and orofacial-related disability. Also, to the best of our knowledge, there is no previous study in the literature that investigated the additional effect of PNE program in patients with TMD. This study will help to better understand if PNE will add a significant effect (immediate and at follow-up) to a manual therapy/movement therapy protocol in patients with chronic pain TMD.

In physiotherapy clinical practice it is a common approach to deliver manual therapy + exercises to manage TMD patients. The previous systematic review with meta-analysis showed an effect of manual therapy for pain intensity and disability compared to other interventions [10, 11, 43] or when orofacial manual therapy is associated with neck exercises [9]. However, it is noteworthy a generalized problem of small sample size for the studies conducted in the field. Moreover, no study was conducted to assess the effect of adding a psychosocial intervention to manual therapy and therapeutic exercise for TMD patients.

In order to put in to practice a biopsychosocial approach in the chronic pain field, it is mandatory the conciliation of education strategies focused on psychosocial factors and interventions related to movement therapy. There are several systematic reviews showing promising effects of PNE for chronic pain patients [17, 44]. A meta-analysis showed that the addition of PNE to other interventions showed a significant effect on pain intensity, disability, and catastrophizing [17].

In this way, this study will help to bring several contributions to the literature studies available in the literature: (i) the initiative to incorporate PNE to TMD treatments commonly delivered; (ii) the assessment of the effect of PNE on psychosocial outcomes such as kinesiophobia and self-efficacy in TMD patients; and (iii) the use of both orofacial manual therapy and orofacial and neck exercises to treat patients with chronic TMD.

## Trial status

Protocol number: NCT03926767. Registered April 29, 2019. Recruiting: Study start

date: April 24, 2019. Study completion date: February 2022.

## Data Availability

The datasets used and/or analyzed during the current study are available from the corresponding author on reasonable request.

## Abbreviations

TMD: Temporomandibular disorders
PNE: Pain neuroscience education
HADS: Hospital Anxiety and Depression Scale
NPRS: Pain Numeric Rating Scale
PCS: Pain Catastrophizing Scale
CF-PDI: Craniofacial Pain and Disability Inventory
PSEQ: Pain Self-Efficacy Scale
TSK/TMD: Tampa Scale for Kinesiophobia for Temporomandibular Disorders
NPQ: Neurophysiology of Pain Questionnaire
GPE: Global Perceived Effect
EARS: The Exercise Adherence Rating Scale
